# Tramadol vs dexmedetomidine for emergence agitation control in pediatric patients undergoing adenotonsillectomy with sevoflurane anesthesia: prospective randomized controlled clinical study

**DOI:** 10.1186/s12871-017-0332-4

**Published:** 2017-03-11

**Authors:** Nurdan Bedirli, Mehmet Akçabay, Ulku Emik

**Affiliations:** Department of Anesthesiology, Gazi University Medical School, Kumeevler cad., Siyasal sit., No:44, 06810 Cayyolu, Ankara Turkey

**Keywords:** Tramadol, Dexmedetomidine, Pediatric recovery

## Abstract

**Background:**

This study was designed to compare the efficacy of an intraoperative single dose administration of tramadol and dexmedetomidine on hemodynamics and postoperative recovery profile including pain, sedation, emerge reactions in pediatric patients undergoing adenotonsillectomy with sevoflurane anesthesia.

**Methods:**

Seventy-seven patient, aged 2–12, undergoing adenotonsillectomy with sevoflurane anesthesia was enrolled in this study. Patients were randomly assigned to receive either intravenous 2 mg/kg tramadol (Group T; *n* = 39) or 1 μg/kg dexmedetomidine (Group D; *n* = 38) after intubation. Heart rates (HR), mean arterial pressure (MAP) were recorded before induction, at induction and every 5 min after induction. Observational pain scores (OPS), pediatric anesthesia emergence delirium (PAED) scores, percentage of patients with OPS ≥ 4 or PAED scale items 4 or 5 with an intensity of 3 or 4, and Ramsay sedation scores (RSS) were recorded on arrival to the postoperative care unit (PACU) and at 5, 10, 15, 30, 45, 60 min. Extubation time and time to reach Alderete score > 9 were recorded.

**Results:**

Dexmedetomidine significantly decreased the HR and MAP 10 and 15 min after induction; increased the RSS 15, 30 and 45 min after arrival to PACU. OPS and PAED scores and percentage of patients with OPS ≥ 4 or PAED scale items 4 or 5 with an intensity of 3 or 4 in both groups did not show any significant difference. Extubation time and time to have Alderete score > 9 was significantly longer in Group D.

**Conclusion:**

Both tramadol and dexmedetomidine were effective for controlling pain and emergence agitation. When compared with tramadol intraoperative hypotension, bradycardia and prolonged sedation were problems related with dexmedetomidine administration.

**Trial registration:**

Retrospectively registered, registration number: ISRCTN89326952 registration date: 14.07.2016

## Background

Adenotonsillectomy in childhood is a common ambulatory surgical procedure requiring general anesthesia, effective pain control, and fast tract recovery [1]. Postoperative period following adenotonsillectomy is often challenging and maintaining effective pain control is important to decrease the incidence of the emergence agitation, dehydration, and hemorrhage from healing surgical wounds [1–3]. The choice of analgesic agents influences the occurrence of complications such as postoperative nausea and vomiting (PONV), emergence agitation, and cardiorespiratory complications [4].

Sevoflurane is a preferred inhalational anesthetic for both general anesthesia induction and maintenance in children because it is characterized with rapid onset and offset, less irritation to the airway [5]. However, sevoflurane is associated with a high incidence of emergence agitation which may delay patient discharge from the hospital [5–8].

Emergence agitation is a frequent complication in children undergoing otorhinolaryngologic surgery under general anesthesia, with an estimated incidence of 18–80% [8]. Emergence agitation has the risks of self-injury, delayed discharge, extra nursing care, family dissatisfaction, and increased cost [9].

Dexmedetomidine, a highly selective α-2 receptor agonist and commonly used in adult anesthesia and intensive care [10–12]. Studies evaluating the intraoperative Dexmedetomidine showed that opioid consumption and pain intensity incidence reduced [1, 2, 12]. Studies in pediatric patients evidenced that Dexmedetomidine is also beneficial in the pediatric perioperative period, it’s anxiolytic, sedative, and analgesic properties is beneficial for emergence agitation control [1, 2, 9, 13]. Bradycardia and decrease in blood pressure are common unwanted sympatholytic side effects associated with dexmedetomidine administration and even sinus arrest has been reported with dexmedetomidine [14, 15]. These hemodynamic side-effects of dexmedetomidine limit its application at anesthesia practice of pediatric patients [9, 12].

Tramadol, a derivate of aminocyclohexanol, has similar analgesic action compared with the opioids and has potential advantages including a long duration of action, rapid recovery, and limited respiratory depressant effects [16, 17]. Tramadol may offer a choice for treatment of pain after tonsillectomy [18]. Tramadol has been shown to reduce postoperative analgesic requirements when given intraoperatively [19]. Although the overall evidence regarding tramadol for postoperative pain in children is currently low, studies demonstrated that intraoperative administration of tramadol provided effective postoperative analgesia with fast tract recovery [20].

This study was designed to evaluate the efficiency of dexmedetomidine and tramadol for controlling postoperative pain, emergence agitation, and intraoperative hemodynamic side effects in patients undergoing ambulatory adenotonsillectomy.

## Methods

The study was approved by local ethics committee, written informed consent from the parents/guardians was obtained, and the study performed in accordance with the Declaration of Helsinki. Eighty patients, ASA physical status I–II aged 2–12, undergoing adenotonsillectomy were enrolled in this prospective, randomized, double blinded study. Patients with the history of developmental delay, cardiac disorder, psychological disorder, epilepsy, and allergy to study medications were excluded from the study. Patients did not have any premedication.

Standard monitoring included electrocardiogram, noninvasive blood pressure, pulse oximetry, and inspiratory and expiratory gas concentrations. Anesthesia was induced with 8% inspired sevoflurane and 60% nitrous oxide in oxygen by facemask. After induction an intravenous catheter was inserted and 1 μg/kg intravenous (iv) fentanyl was given as bolus and rocuronium 0.6 mg/kg was administered to facilitate tracheal intubation.

Patients were assigned to one of two groups according to a computer generated random numbers table. After intubation patients in dexmedetomidine group (Group D, *n* = 38) received 1 μg/kg dexmedetomidine and the patients in tramadol group (Group T, *n* = 39) received 2 mg/kg tramadol. The study drugs were diluted in saline as follows: dexmedetomidine 20 μg/ml (2 ml of dexmedetomidine and 8 ml of saline) and tramadol 20 mg/ml (4 ml of tramadol and 6 ml of saline) and administered as a single iv dose over a 10 min’ period. The anesthetist who administered the study drugs was blinded to the patient group and excluded from the perioperative assessment of the patient. Heart rate (HR), mean arterial pressure (MAP), peripheral oxygen saturation (SpO_2_), and minimum alveolar concentration (MAC) were recorded before induction (baseline), at induction and every 5 min after induction during the procedure.

For anesthesia maintenance patients received 2% sevoflurane in 60% N_2_O and 40% oxygen with controlled ventilation to maintain normocapnia. In both groups 25% increase in HR and MAP with respect to the value before anesthesia induction and sustained for 5 min was considered as tachycardia and hypertension respectively and treated with 0.5 μg/kg fentanyl. Likewise, 25% decrease in HR and MAP is defined as bradycardia and hypotension respectively. Atropine 0.01 mg/kg intravenously for bradycardia and 10 ml/kg normal saline solution for hypotension was administered to the patients. Intraoperative dexamethasone 0.5 mg/kg (maximum 10 mg), ondansetron 0.1 mg/kg and antibiotics were administered to all patients according to institutional practice for tonsillectomy. Normal saline solution was administered for fluid management to all the patients.

Tonsillectomies were performed by two surgeons using extracapsular electro-cautery dissection technique. When hemostasis was achieved, anesthetic gases were discontinued and neuromuscular blockade was reversed with neostigmine 0.05 mg/kg and atropine 0.02 mg/kg iv. The trachea was extubated when patients had eye opening, purposeful movement, or response to command and the patients were transferred to post anesthesia care unit (PACU). The duration between the termination of anesthetic gasses and the extubation was defined as ‘extubation time’.

In PACU the intensity of pain was assessed by using a modified Hannallah pain score [21] – an observational pain score (OPS). Moreover, pediatric anesthesia emergence delirium (PAED) scale [22], Ramsay sedation score (RSS) [23], HR, MAP, SpO_2_ were measured and recorded on arrival to PACU and 5, 10, 15, 30, 45, 60 min after arrival to PACU. Besides, any adverse effects such as vomiting, airway obstruction, laryngospasm or bronchospasm were recorded. All these measurements and recordings were done by the same doctor who was blind to the group of the patient.

The number of patients who presented with OPS ≥ 4 or PAED scale items 4 *‘the child is restless’* or 5 *‘the child is inconsolable’* with an intensity of 3 (very much) or 4 (extremely) were recorded and 0.05 mg/kg morphine as rescue analgesic were administered to these patients. Patients who had an Alderete score > 9 discharged from PACU to surgical ward. The duration between acceptance to PACU and discharge from PACU to surgical ward was defined as ‘time to reach Alderete score > 9’.

Duration of surgery and anesthesia, extubation time, need for morphine, the incidence of vomiting during the 60 min period, and time to reach Alderete score > 9 was recorded.

### Statistical analyses

Statistical analyses were performed using statistical package (SPSS 11.0 for windows, SPSS Inc, Chicago). Data are presented as mean and standard deviation, median and range as appropriate. The primary end point of the study was the need for rescue morphine of the patients in PACU. The secondary outcomes were the degree of sedation of the patients in PACU, incidence of postoperative vomiting, and the time to reach Alderete score of >9. The number of patients in each group was determined by a power calculation based on the results of Qlutoye AO et al. [2]. It was calculated that 35 patients needed per group assuming an α risk of 0.05 and a β risk of 0.10 and mean difference of 50%. To account for exclusion of some patients, we enrolled 40 patients in each group. Demographic data compared using the Student’s *t-test*. MAP, HR, SpO_2_, MAC data were analyzed using two-way repeated measures analysis of variance (ANOVA). OPS, PAED, and PAED score were expressed as median values and compared with Mann-Whitney *U* test. Statistical significance was accepted when the *P* value was less than 0.05.

## Results

**Fig. 1:**
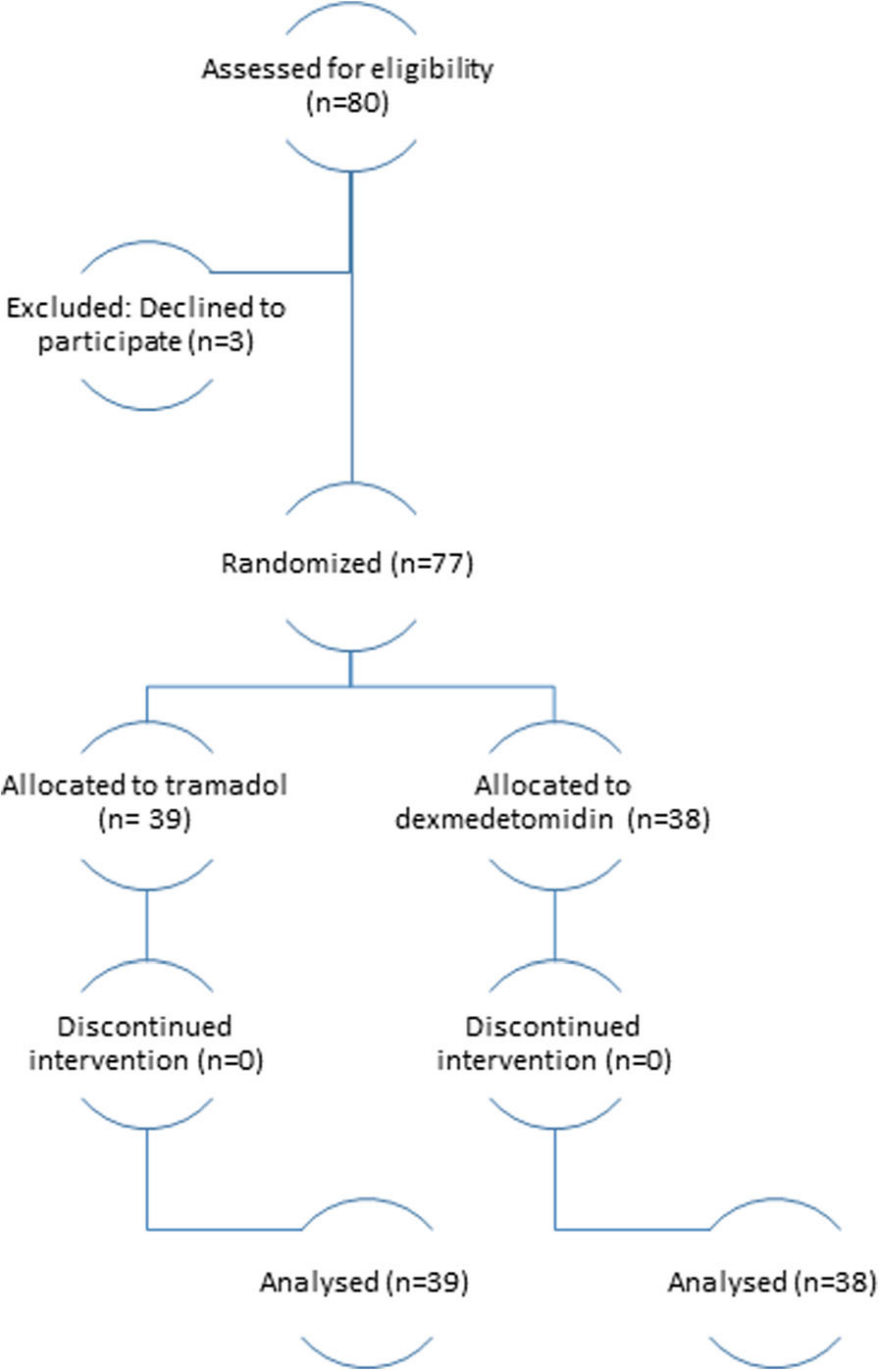
Flow chart of the study

Eighty patients enrolled randomization and seventy-seven were included in the analysis. Three patients were excluded because the parents did not agree to participate (Fig. 1). The two groups were comparable with respect to age, weight, gender, anesthesia time, and surgical time (Table 1). The difference with regard to MAC values of sevoflurane in Group T and Group D was not significant (Table 1, *P =* 0.12). Extubation time and time to have Alderete score > 9 was significantly longer in Group D (6.8 ± 1.7 min and 37.6 ± 5.4 min) when compared to Group T (3.2 ± 0.6 min and 15.2 ± 4.7 min) (Table 2 *P =* 0.0012 and *P =* 0.0013, respectively). The mean dosage of rescue morphine requirement in PACU was 0.07 ± 0.01 mg/kg in Group T and 0.06 ± 0.02 mg/kg in Group D and the difference was not significant (Table 2, *P =* 0.16). In addition, there were no significant difference in the incidence of patients requiring rescue morphine between the two groups (Table 2, *P =* 0.17). Comparison of the incidence of vomiting between the groups did not show any significant difference during postoperative period (Table 2, *P =* 0.31). None of the patients exhibited with signs of laryngospasm or bronchospasm in PACU.

**Table 1:** Demographic and Clinical Data

	Group T (*n* = 39)	Group D (*n* = 38)	*P* value
Age (years)	8.4 ± 2.1	6.7 ± 3.1	0.06
Weight (kg)	28.3 ± 3.7	27.1 ± 2.7	0.16
Gender (M/F)	27/13	21/19	0.26
Duration of surgery (minutes)	38.7 ± 7.4	40.2 ± 5.2	0.31
Duration of anesthesia (minutes)	40.3 ± 1.3	41.9 ± 1.2	0.36
MAC values	1 ± 0.2	0.9 ± 0.2	0.12

Data are expressed as number of patients and mean ± SD

**Table 2:** Data in PACU

	Group T (*n* = 39)	Group D (*n* = 38)	*P* value
Rescue morphine			
•Mean dosage (mg/kg)	0.07 ± 0.01	0.06 ± 0.02	0.16
•Incidence (number of patients)	19	17	0.17
Incidence of vomiting (number of patients)	6	4	0.31
Time to reach Alderete Score > 9 (minutes)	15.2 ± 4.7	37.6 ± 5.4	0.0013^*^
Extubation time	3.2 ± 0.6	6.8 ± 1.7	0.0012^*^

Data are expressed as number of patients and mean ± SD; ^*^ *P* < 0.05, Group T was compared to Group D

**Fig. 2:**
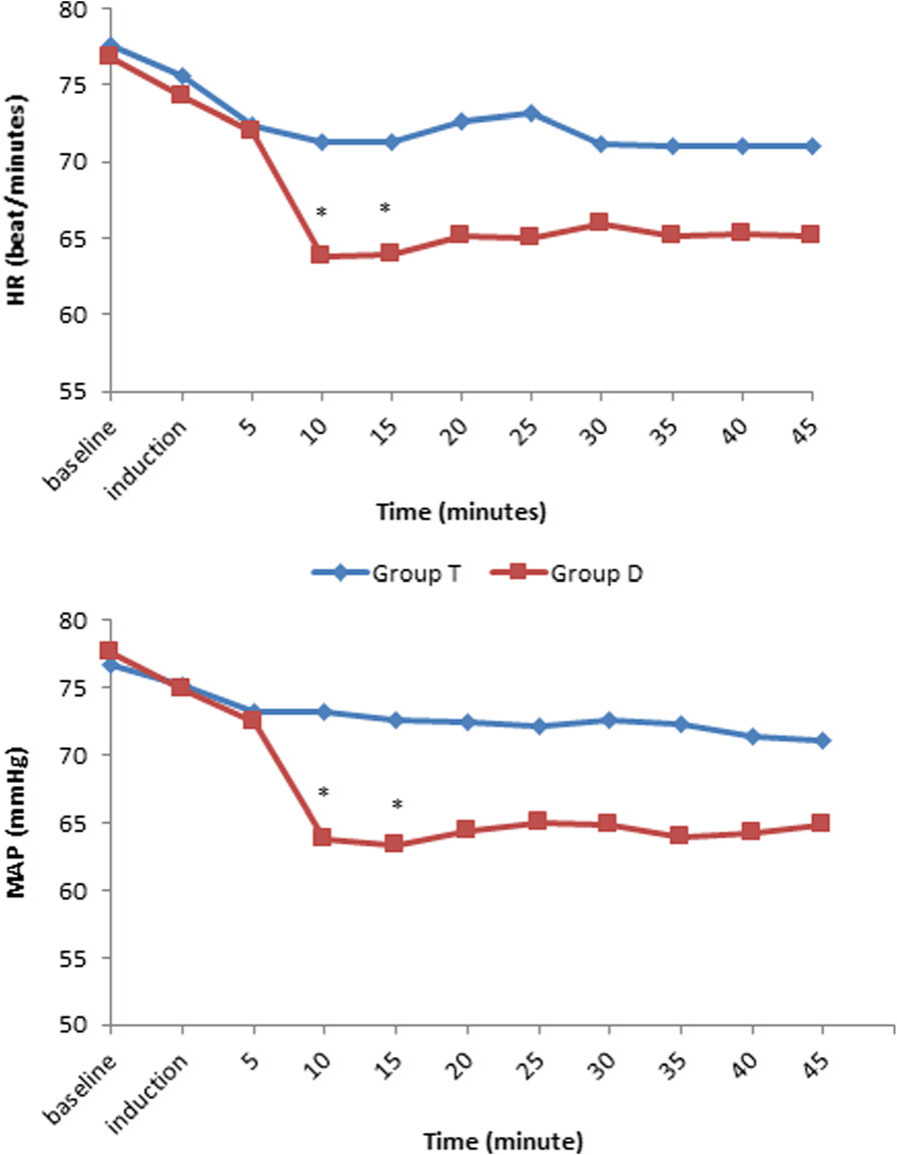
Intraoperative Mean Arterial Pressure (MAP) and Heart Rate (HR) values of the groups. ^*^ *P* < 0.05, when Group T is compared to Group D

Intraoperative HR and MAP are presented in Fig. 2. When compared to tramadol administration dexmedetomidine decreased the HR and MAP values all through the procedure. In Group D, HR significantly decreased 10 and 15 min after induction (*P =* 0.002 and *P =* 0.001, respectively) and MAP also decreased significantly at the same time points (*P =* 0.001 and *P =* 0.001, respectively). None of the patients required rescue fentanyl for tachycardia or hypertension during the procedure. Postoperative HR and MAP did not show any significant differences between groups (*P >* 0.05). SpO_2_ values were ranged between 97 and 100% in both groups throughout the study and there were no significant differences between the groups (*P =* 0.22).

OPS and PAED data are presented in Tables 3 and 4. Both OPS and PAED scores and the number of patients with OPS ≥ 4 and PAED items ≥ 4 with an intensity of 3 or 4 in both groups did not showed any significant difference (*P >* 0.05). Patients in Group D had significantly higher sedation scores 15, 30 and 45 min after arriving to PACU compared to patients in the Group T (*P =* 0.003, *P =* 0.002, *P =* 0.002, respectively, Table 5).

**Table 3:** Observational Pain Score (OPS) Evaluation

		Arrival	5 min	10 min	15 min	30 min	45 min	60 min
Group T (*n* = 39)	OPS score	3	3	2	0	0	0	0
		(2–5)	(1–5)	(1–3)	(0–0)	(0–0)	(0–0)	(0–0)
	Number of patients OPS ≥4	5	4	0	0	0	0	0
Group D (*n* = 38)	OPS score	5	5	4	3	1	0	0
		(3–8)	(3–7)	(3–5)	(1–4)	(0–2)	(0–0)	(0–0)
	Number of patients OPS ≥4	4	3	2	0	0	0	0

Data are expressed as median (IQR 25–75%) and the number of the patients

**Table 4:** Pediatric Anesthesia Emergence Delirium Scale (PAED) Evaluation

		Arrival	5 min	10 min	15 min	30 min	45 min	60 min
Group T (*n* = 39)	PAED score	14	11	10	6	3	2	2
		(3–17)	(3–13)	(0–11)	(0–10)	(0–5)	(0–5)	(0–5)
	Number of patients PAED items 4 or 5 with an intensity of 3 or 4	3	2	0	0	0	0	0
Group D (*n* = 38)	PAED score	15	8	3	2	2	1	1
		(0–17)	(0–15)	(0–6)	(0–5)	(0–5)	(0–5)	(0–5)
	Number of patients PAED items 4 or 5 with an intensity of 3 or 4	3	3	0	0	0	0	0

Data are expressed as median (IQR 25–75%) and number of the patients

**Table 5:** Ramsay sedation scores of the patients

	Arrival	5 min	10 min	15 min	30 min	45 min	60 min
Group T (*n* = 39)	3	3	3	2	2	2	2
	(1–3)	(1–3)	(2–3)	(2–2)	(2–2)	(2–2)	(2–2)
Group D (*n* = 38)	4	4	4	4^*^	4^*^	4^*^	2
	(1–5)	(1–5)	(2–5)	(2–5)	(2–4)	(2–4)	(2–2)

Data are expressed as median (IQR 25–75%). ^*^ *p* < 0.05; The difference was significant when Group T was compared to Group D

## Discussion

In this study, dexmedetomidine or tramadol was administered intraoperatively to children scheduled for adenotonsillectomy under sevoflurane anesthesia and the two drugs were compared with regard to intraoperative hemodynamic stability, postoperative pain management, emergence agitation control, and residual sedation levels.

Pain after tonsillectomy is often intense and long lasting and post tonsillectomy analgesia requires a drug that provides analgesia without respiratory depressant effect that can be used in an ambulatory setting [24]. Dexmedetomidine offers some of these desired characteristics by providing relatively fast onset of sedative properties paralleling natural sleep, with minimal respiratory depression [13, 25–27]. But Dexmedetomidine administration may result in hypotension, bradycardia and even asystole in both children and adults and mostly these side effects have been reported when dexmedetomidine was used as rapid bolus dosages [1, 14, 15, 28]. In this study, dexmedetomidine was administered only as a single bolus dosage by slow intravenous injection but still we had to manage these side effects in our patients. The main problems associated with dexmedetomidine administration in our study were intraoperative hypotension, bradycardia, prolonged extubation time, and residual sedation which caused prolonged PACU stay.

The efficacy of tramadol for the management of moderate to severe postoperative pain has been demonstrated in both inpatients and ambulatory surgery patients. Most importantly, unlike other opioids, tramadol has no clinically relevant effects on respiratory or cardiovascular parameters [29]. Tramadol is shown to be effective in relieving moderate to severe pain in children [30], while exhibiting less respiratory depression and sedation [31]. Still, Tramadol is related with side effects and the most common side effects are nausea-vomiting, dizziness, drowsiness, and constipation [32]. In the pediatric age, the reported incidence of adverse events is similar to that observed in adults and increases with chronic use [33].

Despite the lack of pediatric labeling of both dexmedetomidine and tramadol, both drugs are used worldwide to treat children. In the present study, the dosage of the dexmedetomidine and tramadol was selected based on the previous studies reported in the literature [1, 2, 17, 33]. Olutoye OA et al. [2] reported that dexmedetomidine 1 μg/kg administration to pediatric patients undergoing adenotonsillectomy had the advantages of an increased time to first analgesic and a reduced need for additional rescue analgesia doses in the PACU. Pestieau SR et al. [34] reported that high-dose dexmedetomidine needed to decrease opioid requirements and provide opioid-free interval after tonsillectomy in the post anesthesia care unit. Bozkurt et al. [33] reported that 2 mg/kg iv dosage of tramadol is the best for analgesic action with minimal side effects. Therefore, in this study we investigated dexmedetomidine 1 μg/kg and tramadol 2 mg/kg administered as slow intravenous injection.

Pain, sore throat, laryngeal edema and airway obstruction may occur after adenotonsillectomies and are the probable etiological factors of emergence agitation. In the present study, dexamethasone and fentanyl were administered to all patients for the prevention of laryngeal edema and pain. Fentanyl administrated at induction to all the patients and planed as intraoperative rescue analgesic but none of our patients needed intraoperative rescue analgesic and dexamethasone was the drug chosen for the prevention edema for both groups. Evidently both groups benefited from the decrease in incidence of the emergence agitation but this did not affect the comparison of the groups because all the patients in both groups received the equivalent dosage of dexamethasone and fentanyl.

There are some limitations in this study. First of all, we did not monitor the depth of anesthesia by BIS. Secondly, the broad range of our patients’ age is limiting factor because age distribution is important in evaluating the postoperative pain and recovery profiles including agitation. Moreover, if there had been a control or placebo group it would be more possible to define the effectiveness of the study drugs. But at this time, failure to provide adequate analgesia in the placebo group would cause serious ethical and clinical problems.

The analgesic action of tramadol is based on a multimodal mechanism of action that involves activation at the opioid receptor as well as inhibition of norepinephrine and serotonin reuptake in the central nervous system [17, 33]. Chu YC et al. [17] have demonstrated that, intraoperative tramadol caused earlier awakening from general anesthesia without prolonged sedation and earlier tracheal extubation in the immediate postoperative period. However, the administration of tramadol for postoperative analgesia also may have an adverse effect on the incidence of nausea and vomiting [17]. In this study, tramadol was effective in managing pain and emergence agitation with apparent lack of prolonged sedation in the early postoperative period. Moreover, extubation times of the patients who received tramadol were shorter and vomiting incidence was similar in the two groups. This data may be the result of routine administration of ondancetron and dexamethasone besides intraoperative administration of tramadol or dexmedetomidine to our patients.

## Conclusion

In this study, we have demonstrated that in pediatric patients undergoing ambulatory adenotonsillectomy both dexmedetomidine and tramadol were effective in controlling postoperative pain and emergence agitation but in comparison to tramadol dexmedetomidine administration resulted in higher incidence of, intraoperative bradycardia, hypotension, postoperative prolonged sedation, and prolonged PACU stay.

## Abbreviations

ANOVA: Analysis of variance
HR: Heart rate
Iv: Intravenous
MAC: Minimum alveolar concentration
MAP: Mean arterial pressure
OPS: Observational pain score
PACU: Post anesthesia care unit
PAED: Pediatric anesthesia emergence delirium
RSS: Ramsay sedation score
SpO2: Peripheral oxygen saturation
